# What motivates start-up entrepreneurs? Exploring the role of human values in success

**DOI:** 10.1371/journal.pone.0312944

**Published:** 2024-12-13

**Authors:** Eamon Curtin, Gabriel Lins de Holanda Coelho, Paul H. P. Hanel

**Affiliations:** 1 University College Cork, Cork, Ireland; 2 University of Essex, Colchester, United Kingdom; Sichuan Agricultural University, CHINA

## Abstract

This study explores how entrepreneurs perceive success, the influence of their values on this perception, and the association between values and success. We surveyed 96 Irish entrepreneurs to capture their definitions of success, their own values, their perceptions of a successful entrepreneur’s values, and various success metrics. We coded the qualitative responses regarding what success means to them using Schwartz’s (1992) model of human values. We found different and partly conflicting interpretations of success, suggesting that entrepreneurial success is multidimensional. While many participants interpreted success as related to achievement and power, others interpreted it as related to universalism and benevolence, going beyond past research that defined entrepreneurial success as primarily based on self-enhancement motives. Further, entrepreneurs in our sample valued conformity, tradition, and security less than the average person in Ireland, but stimulation and self-direction more. Interestingly, entrepreneurs’ values were unrelated to the understanding of success and the actual success of entrepreneurs, as measured by turnover and the number of staff. This is an encouraging finding because it suggests that people can become successful entrepreneurs independently of their values. Together, our findings provide new insights into how entrepreneurs conceptualize success, revealing both self-enhancing and self-transcending perspectives.

## Introduction

Human values are abstract ideas that embrace our motivations or goals, and guide our behavior [1, 2]. Researchers have been studying values across the social sciences and humanities [3], and their role in predicting attitudes and behavior has helped to increase their popularity [4–7]. This popularity is evidenced by the multidisciplinary application of human values over the years, with researchers from different fields demonstrating that values predict relevant outcomes such as environmental issues [8], educational motivators [9], and even behavioural responses to the Covid-19 pandemic restrictions [10]. Not surprisingly, the study of values within organizations has also gained prominent attention over the past decades. Researchers are interested in how values can help understand the underlying mechanisms that motivate employees to rise and succeed in their roles and consequently lead the company to thrive [11, 12].

However, despite this growing interest, one of the less researched areas is the extent to which the values of start-up founders are related to the success of their start-ups. Understanding these factors can be vital in effectively using public and private resources for start-up support. It can inform the decision-making for organizations running start-up incubation programs, accelerator programs, allocating start-up grants, and for angel investors, venture capitalists, and private equity firms making investments in start-ups.

Our research focuses on providing an exploratory understanding of the organizational aspects of human values and their links to success. More specifically, we test whether the values of start-up entrepreneurs from an incubator in Ireland and other entrepreneurs are associated with quantitative measures of success (e.g., turnover, number of staff), their qualitative understanding of success, how they perceive the values of successful entrepreneurs, and whether their values are different to those of the general population.

### Human values within organizations

**Fig 1:**
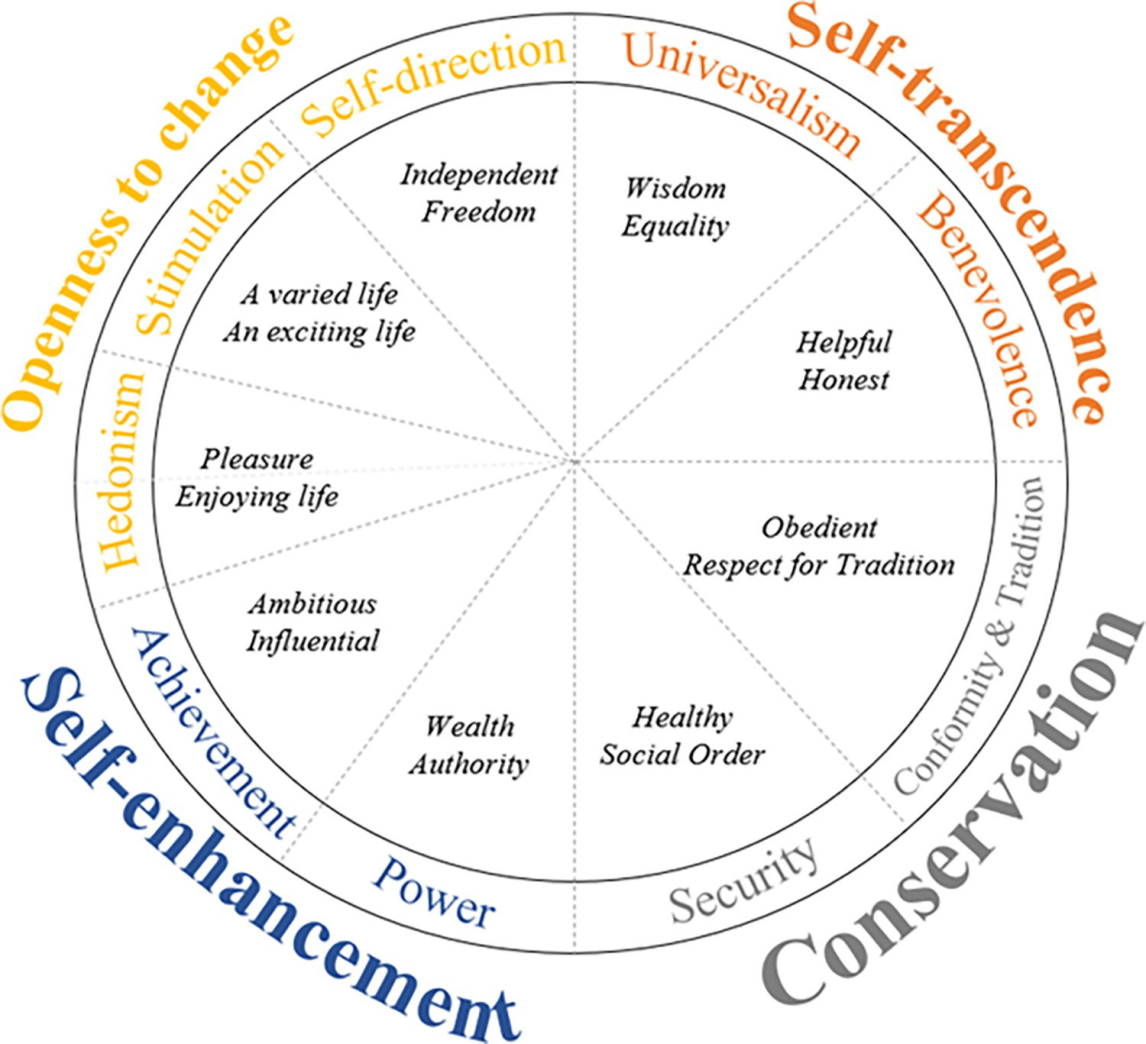
Schwartz`s model of human values (in italic, examples of values). Image from (Coelho et al., 2019) [19].

The Theory of Basic Human Values is the most well-known and cited model of values in psychology, with robust structural evidence from over 80 nations worldwide [2, 13]. Schwartz’s proposed a circular organization of human values, with 57 values (e.g., ambitious, equality), spread across ten value types (e.g., power, self-direction; cf. Fig 1). Some values have similar underlying motivations and are more related to each other. For instance, power and achievement values reflect a self-enhancement motivation, which focuses on the self; therefore, they are positively related. In contrast, benevolence values reflect a self-transcendent motivation, focusing on other people, and therefore, are unrelated or negatively related to self-enhancement values. Individuals with higher power values might seek authority over others or have personal monetary gains in their company. In contrast, individuals who endorse universalism values might attempt to develop an equal environment among their peers. The other dimension of the model contrasts openness values (e.g., daring, freedom, creativity) with conservation values (e.g., security, obedience, religiosity).

Several studies researched the role of values for entrepreneurs. For instance, Gorgievski et al. [14] assessed the associations between personal values and business owners’ understanding of success in 150 Dutch small business owners. Their results showed that business growth, profitability, and innovativeness are guided by power and achievement values (self-enhancement motivation), whereas having satisfied stakeholders and a better work-life balance are guided by benevolence and universalism values (self-transcendent motivation). Moreover, Morales et al. [15] assessed the cultural contexts where individuals are most likely to be entrepreneurs across 35,000 respondents from 28 European countries. The authors found that individuals who prioritize achievement and power values and prioritize seeking stimulation and self-direction (openness to change motivation) are more likely to be self-employed than others. They also assessed the influence of culture, with individual values being more relevant to explain entrepreneurship if societies do not display high entrepreneurial cultural values (low mastery and egalitarianism). A more recent study found that career preferences related meaningfully to values [16]. For example, self-enhancement values were positively correlated with career preferences for income and prestige, whereas self-transcendence correlated positively with preferences for caring professions as well as with working with animals and plants. Overall, these results highlight that associations between values and entrepreneurship exist. However, despite those findings, a closer look is needed to provide more in-depth inferences about the link between values and entrepreneur success.

### The present research

In the present study, we examined for the first-time what success means to entrepreneurs, whether their understanding of success can be mapped onto Schwartz’s values, how their personal and perceived values of relate to their business performance, and whether these values differ from the general population. More specifically, using a sample from start-up incubators in Ireland and other entrepreneurs, we asked participants to reflect on their understanding of success, rate their personal values and how they perceive the values of a successful entrepreneur, and provide information regarding their start-ups. Additionally, we identify the unique value profile of entrepreneurs by comparing the values of entrepreneurs with a representative sample of Irish people. Since being an entrepreneur is associated with risk taking, but also allows them to be more independent, we speculate that entrepreneurs score higher in stimulation and self-direction. This is because previous research established that stimulation is associated with risk taking behaviours [17, 18, 20]. Further, self-direction is associated with independent and novel behaviour [17, 18] as well as the tendency to enjoy thinking [21], which in turn is positively associated with entrepreneurial intentions [22]. Conversely, because security, conformity, and tradition are about maintaining the status quo, we expect entrepreneurs to score lower on those values compared to the average person living in Ireland.

Further, in line with similar research discussed above, we predict that being successful as an entrepreneur is positively associated with achievement and power, since these values express self-enhancing motivations. We do not make any predictions regarding universalism and benevolence, because whether these value types are correlated with success might depend on the sector entrepreneurs are working in [14]. For example, if someone is working in the caring or environment sector, universalism and benevolence are likely positively associated with success, perhaps also because their values fit with their work [23].

Importantly, our focus on perceived values goes further beyond past research in organization science and builds on research from personality and social psychology. For example, Sanderson et al. [24] found that British citizens who perceived British people valuing self-transcendence values reported more positive attitudes toward civic engagements. Further, people who perceive their group to value biospheric values more show more robust pro-environmental engagement [25]. However, how other people are perceived is often biased. Our design allows us to test whether entrepreneurs perceive the values of other entrepreneurs in a biased way. Such a biased perception was already found within the general public [26]. A biased perception (e.g., perceiving successful entrepreneurs’ values to be different from their actual values) might lead to poorer perceived fit and, therefore, higher intentions to change jobs. If the perception turns out to be biased, this might help to design interventions in which the biased perceptions are rectified to reduce dropouts. The survey, data, and the R-code to reproduce our analyses can be found on https://osf.io/vsnuq/?view_only=e2a6fd75d668401497717688a6de2e29.

#### Methodological choices and why this method is used

The use of a questionnaire to measure human values is very common in the literature, as are all of the various questionnaires that were proposed to measure Schwartz’s [2] model of human values [27–30]. Value ratings obtained through those questionnaires were correlated with a range of outcomes including behaviour [7, 31, 32], attitudes [6, 33], personality traits [34, 35], and various well-being indicators [36, 37]. The way we measure perceived values is adapted from previous research which found that perceived values can influence pro-social engagement [24], pro-environmental behaviour [25], and prejudice [38]. To measure how our participants understand success, we asked them an open ended question which was adapted from previous research that investigated how people across three countries understand (i.e., exemplify or understand) various human values [39]. In short, all of our key measures have successfully been used in previous research, but not been combined which allows us to get a better understanding of human values.

## Method

### Participants and procedure

Ninety-six entrepreneurs participated in the study (64 men, 30 women, and two preferred not to say). Their mean age was 39.09 years (*SD* = 11.66). The turnover of their companies is listed in Table 1. Sixty-five were self-employed, whereas 30 were not self-employed. Participants were working in a range of sectors. The business and finance sector (n = 25) and health and medicine (17) were the most frequently mentioned. At the time of data collection, the average number of people working in each company was 22.54 (*range*: 0–400, *SD* = 63.87). Participants were informed that their participation is entirely voluntary and that they have the right to withdraw at any stage up to the point of data submission, prior to obtaining written consent. Data were collected between 3 April 2022 and 7 June 2022. Data were collected online. The design was cross-sectional. All procedures performed in this study involving human participants were in accordance with the 1975 Helsinki Declaration. This study was covered by ethics approval at one of the author’s institutions.

**Table 1:** Turnover in 2020.

*Turnover*	*Frequency*
*< €100*,*000*	*50*
*€100*,*000–250*,*000*	*12*
*€250*,*000–1*,*000*,*000*	*6*
*€1*,*000*,*000–5*,*000*,*000*	*10*
*> €5*,*000*,*000*	*14*
*Total*	*92*

To test whether entrepreneurs’ values would differ from those of other people in Ireland, we used data from a representative sample of 2,219 people living in Ireland from the European Social Survey, wave 9. This data was mainly collected in 2019 (*M*_age_ = 52.34, *SD* = 17.69, 1161 women, 1055 men). Participants completed the same value measure as participants in our sample, which is described below.

### Materials

First, we asked participants to think about what success means in their personal opinion and write a few lines explaining it to us. We highlighted that this question had no evaluative character and no answer would be considered right or wrong. This measure was adapted from Hanel et al. [39].

Next, participants completed a 21-item version of the Portrait Value Questionnaire [28], which measures each of Schwartz’s [2] 10 value types with 2–3 items. Participants were instructed to rate each item on how well it described them. Example items include “*I think it is important that every person in the world be treated equally*. *I believe everyone should have equal opportunities in life*.” (universalism) and “*Being very successful is important to me*. *I hope people will recognize my achievements*.” (achievement). Responses were given on a 6-point scale ranging from 1 (Does not describe me at all) to 6 (Describes me very well).

Participants then completed the same questionnaire again but were instructed to “rate how much they [the items] describe a *successful entrepreneur*. That is, an entrepreneur who does something meaningful for others but at the same time is also financially successful. In other words, we are interested in what *characterizes a successful entrepreneur in your view*.” This questionnaire was adapted from several previous studies that measured perceived values such as the values of people who are living in the same country or city [26, 40–42].

Finally, we asked participants a range of questions about their motivation and expectations surrounding their work to explore whether they are also related to success: “How motivated are you to increase your turnover to this level?”, “All things considered, how has your start-up performed compared to your initial expectations?”, and “How strongly does your personal happiness depend on the success of your start-up?”. Those items were developed by us.

## Results

### What is success?

First, we coded participants’ answers regarding their understanding of success. More specifically, we used Schwartz’s value types to categorize their answers. We used Schwartz’s model because many behaviors can be mapped onto the value quasi-circumplex [4, 17, 43]. For instance, some participants point out that success is about “*Being satisfied with work everyday*, *and enjoying your work and personal life*” or “*Being happy with one’s life*” as a perception of success that aligns with **hedonistic** values. Other participants emphasized that success is “*The realization of a worthwhile goal*” or “*Making an impact on as many people’s businesses and lives as possible in my area of expertise*”, aspects that are common to **achievement** values, or “*Having enough money to not worry about bills*”, emphasizing **power** values. Note that some answers referred to more than one value type. For instance, one participant believed success means “*Having a positive impact on the people that surround you*. *Creating a better environment/working area for your team*. *Having the ability to spread knowledge/ financial gain and education others*”, emphasizing characteristics that describe **universalism** and **benevolence** values. Of all participants, 38 provided answers emphasizing achievement values, 33 hedonism, 30 self-direction, 28 power, 27 benevolence, 22 universalism, 14 security, and nine stimulation, whereas none of the answers aligned with tradition or conformity values. The codings and qualitative parts are available as S1 Table. In a series of t-tests, we compared whether the value types in which we categorized participants’ responses were associated with turnover in 2020 or expected turnover in 2023 for all value types with at least ten answers. That is, we compared, for example, participants whose answers were related to achievement values and compared them with participants whose responses did not fall within the achievement category. However, none of the 14 t-tests reached significance, *p*s > .11.

In the next step, we tested whether values and perceived values were related to turnover in 2020, expected turnover in 2023, motivation to increase turnover, number of people working full-time in 2021, and expected number of people working full-time in 2024. However, none of the correlations reached statistical significance, indicating that (perceived) values are unrelated to various success measures in small companies (see S1 Table).

However, some other correlations turned out to be significant. Perhaps non-surprisingly, the number of staff in 2021 correlated with the 2020 turnover, *r*(79) = .51, *p* < .001. Entrepreneurs who reported that their start-ups performed better than they initially expected were aiming for a higher turnover in 2023. Interestingly, whether entrepreneurs believed that their personal happiness depended on the success of their start-up was unrelated to past and expected turnover and the number of staff.

**Fig 2:**
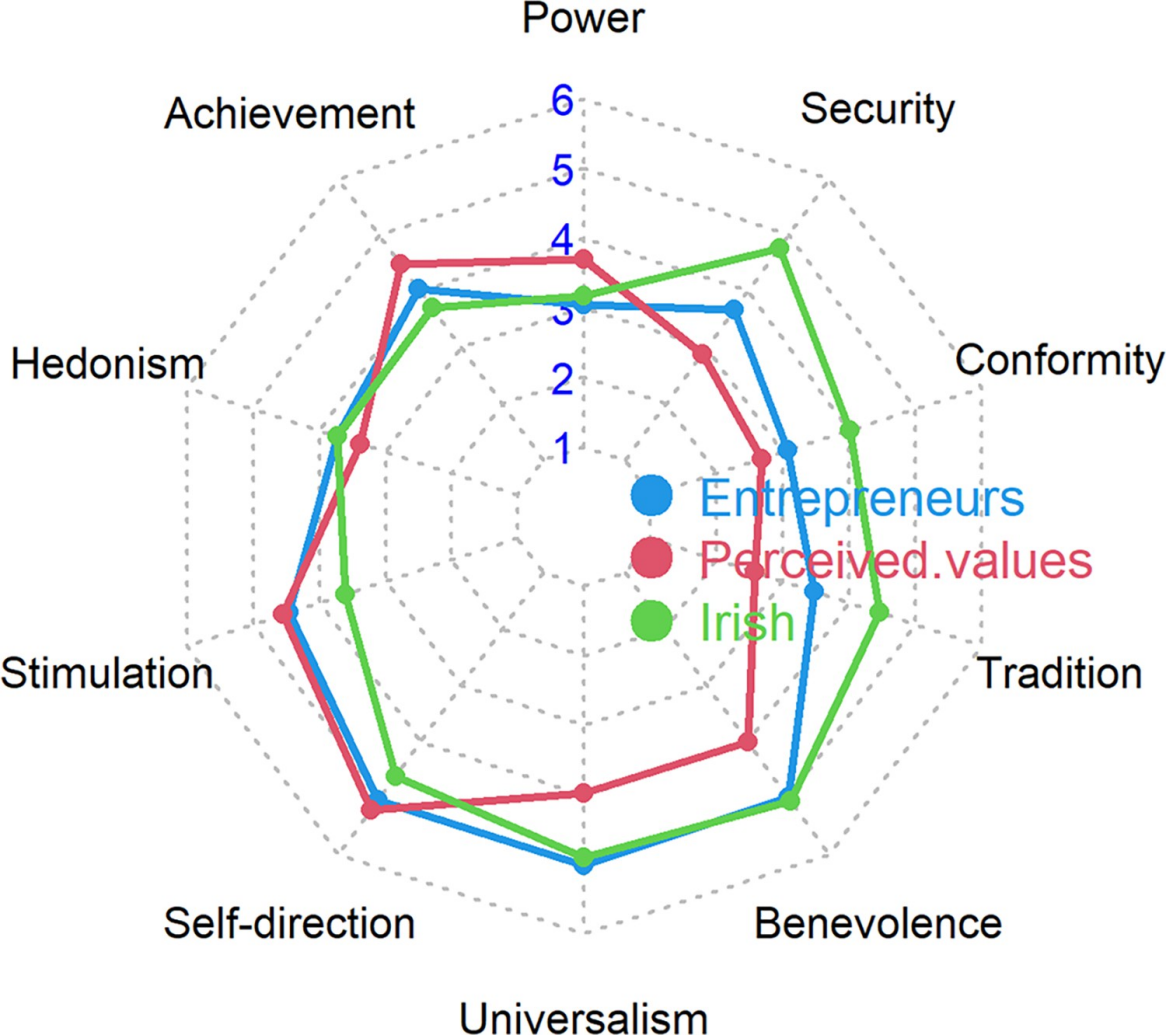
Radarchart of entrepreneurs own values (blue), how they perceive the values of a successful entrepreneur (red), and an Irish representative sample (green). *Note*. 1 indicates a low endorsement of a value, and 6 indicates a high endorsement. “Irish” refers to the average values of people living in Ireland, which is derived from a large representative sample (see Method section for details).

To test whether the value profile of entrepreneurs would be different from the general public, we compared participants’ own values with the perceived values of successful entrepreneurs and a representative sample of the Irish population. Overall, there were many similarities between the participants’ values and the values of an Irish representative sample (Fig 2 and Table 2). Participants endorsed less conformity, tradition, and security than the average person in Ireland. Interestingly, participants perceived successful entrepreneurs as scoring higher than they did themselves on power values but not achievement values and lower on self-direction, universalism, and benevolence.

**Table 2:** Descriptive statistics and comparisons between entrepreneurs’ values and perceived values of a successful entrepreneur as well as between entrepreneurs’ values and an Irish representative sample.

*Value type*	*M (SD)*			*Self-perceived*		*Self-Irish*	
	*Self*	*Perceived*	*Irish*	*t*	*d*	*t*	*d*
*Self-direction*	5.04 (0.87)	5.21 (0.95)	4.61 (0.96)	-1.74	-0.19	4.69***	0.44
*Universalism*	5.02 (0.76)	3.98 (1.08)	4.90 (0.80)	9.55***	1.12	1.48	0.15
*Benevolence*	4.99 (0.77)	4.00 (1.03)	5.05 (0.82)	9.28***	1.09	-0.66	-0.06
*Tradition*	3.48 (0.96)	2.58 (1.02)	4.46 (1.04)	8.84***	0.91	-9.84***	-0.95
*Conformity*	3.08 (1.12)	2.68 (1.05)	4.01 (1.15)	3.61***	0.37	-7.94***	-0.81
*Security*	3.68 (1.21)	2.88 (1.19)	4.77 (1.01)	7.33***	0.67	-8.63***	-1.07
*Power*	3.04 (0.99)	3.70 (1.18)	3.17 (1.07)	-5.42***	-0.61	-1.25	-0.12
*Achievement*	4.05 (1.23)	4.48 (1.18)	3.71 (1.21)	-3.60***	-0.36	2.66**	0.28
*Hedonism*	3.73 (1.04)	3.38 (1.06)	3.73 (1.18)	3.43***	0.34	0	0
*Stimulation*	4.46 (0.99)	4.55 (0.99)	3.61 (1.27)	-0.78	-0.09	8.12***	0.67

Note

*p < .05

**p < .01

***p < .001

Additionally, we performed a series of exploratory analyses. To reduce the likelihood of false positives, we set our alpha threshold for them to .001. First, we compared whether women or men would differ on average on the key entrepreneurial variables. Men reported higher levels of turnover in 2020 than women, *t*(83.44) = 3.96, *p* = .0002, *d* = 0.73, but did not expect significantly higher turnover in 2023. Further, entrepreneurs who felt they positively impacted society did not report higher turnover in 2020 or higher motivation to increase it. Self-employed participants (*n* = 65) did not differ from those who were no longer self-employed (*n* = 30) in their values, how they perceived the values of successful entrepreneurs, turnover, or staff. Also, a series of one-way between subject ANOVAS revealed there were no meaningful differences in values between entrepreneurs who had worked for at least five years (*n* = 34) either in a micro (<10 members of staff), small (10–49 staff), or medium-to-large (>50 staff).

## Discussion

In the present research, we investigated for the first-time what success means to entrepreneurs, whether values predict their success as measured by turnover and the number of staff, how they perceive the values of successful entrepreneurs, and whether entrepreneurs’ values differ from those of the general population. We first discuss the findings and their implications before pointing to some limitations.

In answer to the first question, we found different and partly conflicting motivations for success, suggesting that entrepreneurial success is multidimensional. While many participants interpreted success as related to achievement and power, others interpreted it as related to universalism and benevolence, thereby expanding previous research which focused only on self-enhancement and openness motivations when defining success [44]. At first glance, this seems to contradict Schwartz’s [2] model of human values, which postulates that achievement and power are based on self-enhancement motivations, while benevolence and universalism are on self-transcendence motivations. In other words, they are based on opposing underlying motives. However, understanding success is not directly about values, but related to instantiations [3, 45], which are exemplifier of human values [46]. That the same instantiation can be associated with different values has been established by previous research [39], albeit not yet in a sample of entrepreneurs. Thus, in contrast to common stereotypes that entrepreneurs care primarily about money, we show that self-transcendence motives also drive many. This aligns with recent meta-analytic evidence showing that prosocial motivation can be positively associated with job performance [47, 48].

Contrary to our expectations, values were unrelated to the success of entrepreneurs, at least when considering measures such as turnover and the number of staff. Also, how our participants perceived the values of successful entrepreneurs was unrelated to their own success. Intuitively, this seems surprising in light of evidence that achievement and power values are associated with more positive views towards money [49] and with higher income [50]. However, as discussed above, entrepreneurs understand success differently. That is, their motivation to be successful differs, which is likely why none of the values is linked with the two success measures we used. This might also be because the entrepreneurs were active in many areas, such as business and finances, health and medicine, or social impact. Entrepreneurs working in business and finance are more likely to value achievement and power [51, 52]. Unfortunately, our sample is too small to perform subgroup analyses by business area. If we had focused on only one of the areas, values might again predict success. For example, among all entrepreneurs working in health and medicine-related start-ups, those who place higher importance on benevolence and universalism might be more motivated to increase their turnover. Nevertheless, we believe that not having found an association between values and success is encouraging because it suggests that people can become successful entrepreneurs independently of their values.

However, it is also important to consider the potential influence of the Irish context in these unexpected deviations from a more stereotypical understanding of how values might act in entrepreneurship. For instance, Ireland is known for various policies and initiatives supporting entrepreneurs and small businesses, encouraging them to focus on sustainable growth and collaborations instead of solely on financial gains. This is reflected by the National Entrepreneurship Context Index (NECI), as reported by the 2021 report from the Global Entrepreneurship Monitor [53]. The index covers aspects such as government policies and programs, education at school, commercial and professional infrastructure, and social and cultural norms. Ireland presented a score of 4.7, above the average of 4. The GEM report also estimates that one in seven people in the country will pursue the entrepreneur track over the next three years. However, the impact of the Irish environment is hypothetical and beyond the scope of this study.

We have also found evidence for self-selection or socialization effects: Entrepreneurs in our sample valued conformity, tradition, and security less compared to the average person in Ireland, but stimulation and self-direction more. Longitudinal designs are required to determine whether the mean differences between entrepreneurs and driven by self-selection or socialization effects. Based on previous research on value change [51], we speculate it is a bit of both. Our findings align with other research showing that conservation values such as security or tradition are associated with less independence and risk-taking behavior [18, 54]. In contrast, stimulation and self-direction are associated with the tendency to enjoy abstract thinking (Coelho et al., 2020), which in turn is associated with success [55, 56]. Together, conservation and openness values only predict whether people become entrepreneurs but not whether they are successful.

Further, we found that entrepreneurs perceived the universalism, benevolence, tradition, conformity, security, and hedonism values of a successful entrepreneur compared to their own values on average to be lower, but power and achievement to be higher. This is mostly in line with research from student samples and samples from the general public [26]. In our sample, this effect might be driven by participants being exposed through (social) media to entrepreneurs who have a strong self-enhancement motivation (e.g., increased revenue, profit, and market share). Alternatively, our sample might not have been representative of entrepreneurs in general.

### Implications

Our study has several important implications. Firstly, our research sheds light on the values most closely associated with start-up entrepreneurial success, providing valuable insights for aspiring entrepreneurs and individuals seeking to develop their entrepreneurial skills. We found that entrepreneurs had different and sometimes conflicting understandings of success, if mapped on Schwartz’s [2] value model. While some interpreted success as related to values such as achievement and power, others interpreted it as related to universalism and benevolence. This is important because it challenges common stereotypes that entrepreneurs only care about financial gains. This finding has important implications for entrepreneurship training programs and incubators (such as IGNITE). It suggests that fostering a sense of benevolence and universalism among entrepreneurs could help them fulfill their goals. This aligns with recent meta-analytic evidence showing that prosocial motivation is positively associated with job performance [47, 48]. By encouraging entrepreneurs to focus on values that prioritize the welfare of others, entrepreneurship education programs could promote socially responsible business practices that benefit society. This has also implications for potential investors. It suggests that investors do not need to vet entrepreneurs for their values and understanding of success. Instead, investors could focus on other criteria such as sustainability when deciding where to invest [57].

Secondly, we should highlight the lack of association between values and entrepreneurial success as measured by turnover and the number of staff. This finding is somewhat surprising given previous research that has suggested that values such as achievement and power are associated with more positive attitudes towards money [49] and with higher income [50]. Our findings suggest that entrepreneurs’ understanding of success is complex and not solely focused on financial gain, as seen by our participants’ answers. Such evidence may encourage those interested in becoming entrepreneurs but may not have the typical entrepreneurial mindset associated with values such as achievement and power. It also suggests that people can become successful entrepreneurs independent of their values, which may increase the diversity of entrepreneurs in the business world.

However, despite the findings suggesting the possibility of becoming a successful entrepreneur having a value mindset that works differently from the stereotypical idea of being guided by achievement and power, it is also important to highlight the differences between entrepreneurs’ values compared to the average person in Ireland. Our findings suggest that entrepreneurs are more likely to be risk-takers and are less likely to be tied to tradition or security. These results can have implications for the entrepreneurial ecosystem. If individuals with specific values are more likely to become entrepreneurs, then efforts to encourage entrepreneurship may need to target individuals with those values. For example, suppose stimulation and self-direction values are associated with entrepreneurship. In that case, programs promoting those values may be more effective in fostering entrepreneurship than programs focusing on conformity or tradition. This may be especially relevant for policymakers and educators who aim to promote entrepreneurship as a means of economic development.

Finally, the diversity between entrepreneurs indicates that management practices should accommodate motivational goals (i.e., different values). Specifically, managers and leaders should recognize that employees and team members may differ in their definitions of success and tailor their approaches to support these diverse motivations. For instance, while some entrepreneurs may be driven by financial success and power, others may find fulfillment in making a social impact or fostering well-being of their community.

### Limitations and conclusion

Despite our significant findings, there are several limitations in our study. First, our sample consisted solely of entrepreneurs from micro and small companies in Ireland, therefore limiting our findings’ generalizability. Second, the relatively small sample size limited the possibility of conducting subgroup analyses by business area, which may have revealed more nuanced relationships between values and success measures. Third, our measures of start-up success are incomplete. Customer and staff satisfaction as well as sustainability and reputation among clients are examples of other indicators of success that could be used by future research. Finally, we used only self-reported measures, which could be subject to social desirability and recall biases. Finally, we used only self-reported measures, which could be subject to social desirability and recall biases. However, values are unrelated to socially desirable responses [58, 59]. In future studies, researchers might consider using larger and more diverse samples, incorporating objective measures of success, and triangulating data from multiple sources, such as peer reports to validate the findings.

Together, our study provides novel insights into the values and motivations of entrepreneurs and their understanding of success. Our results challenge common stereotypes by revealing that entrepreneurial success is not solely driven by self-enhancement values like achievement and power, as commonly and stereotypically represented. Instead, entrepreneurs in our sample also reported motivations related to self-transcendence values (universalism and benevolence). We have also found no significant association between values and success as measured by turnover and the number of staff. Such a finding suggests that individuals can become successful entrepreneurs independent of their values, which may foster greater diversity in the entrepreneurial ecosystem.

## Supporting information

S1 Table Correlations between all variables. (DOCX)
