# Supplementary material for: What motivates start-up entrepreneurs? Exploring the role of human values in success
Source: PLoS One. 2024 Dec 13;19(12):e0312944. doi: 10.1371/journal.pone.0312944 (PMC11642908; doi:10.1371/journal.pone.0312944)
Supplement: S1 Table — (DOCX) [file pone.0312944.s001.docx]

Online Supplemental Materials

|  | Turnover2020 | Turnover2023 | Motivated | Staff2021 | Staff2024 | Expectations | Happiness | Age | Gender | SD | UN | BE | TR | CO | SE | PO | AC | HE | ST | SDp | UNp | BEp | TRp | COp | SEp | POp | ACp | HEp | STp |
| --- | --- | --- | --- | --- | --- | --- | --- | --- | --- | --- | --- | --- | --- | --- | --- | --- | --- | --- | --- | --- | --- | --- | --- | --- | --- | --- | --- | --- | --- |
| Turnover2020 | 1 | .76 | .1 | .51 | .46 | .4 | .06 | .54 | -.12 | -.15 | -.24 | -.04 | .07 | .13 | -.2 | 0 | -.09 | -.01 | -.04 | -.1 | -.1 | .1 | .14 | .13 | -.03 | -.01 | .07 | .14 | -.15 |
| Turnover2023 | .76 | 1 | .33 | .49 | .55 | .44 | -.07 | .48 | -.07 | -.23 | -.19 | -.11 | -.02 | -.01 | -.2 | .09 | -.1 | 0 | .08 | -.14 | -.18 | -.02 | .09 | .17 | -.09 | .08 | .04 | .1 | -.17 |
| Motivated | .1 | .33 | 1 | .05 | .1 | .25 | -.06 | -.08 | .07 | .1 | .23 | .04 | .13 | -.07 | .14 | .33 | .24 | .26 | .41 | .02 | .22 | .16 | .1 | .04 | .09 | .23 | .18 | .17 | .2 |
| Staff2021 | .51 | .49 | .05 | 1 | .95 | .31 | -.03 | .37 | -.14 | -.43 | -.33 | -.23 | 0 | .1 | -.2 | -.1 | -.32 | -.12 | .01 | -.2 | -.14 | -.05 | -.09 | .07 | -.17 | .04 | -.06 | .05 | .02 |
| Staff2024 | .46 | .55 | .1 | .95 | 1 | .31 | -.03 | .35 | -.17 | -.36 | -.33 | -.24 | -.03 | .05 | -.18 | -.05 | -.25 | -.13 | .03 | -.17 | -.15 | -.05 | -.07 | .06 | -.17 | .08 | -.02 | .03 | 0 |
| Expectations | .4 | .44 | .25 | .31 | .31 | 1 | -.15 | .13 | .22 | -.12 | -.07 | 0 | -.04 | .13 | -.11 | -.11 | -.12 | -.02 | .07 | .02 | -.04 | .05 | -.01 | .12 | .02 | .02 | .06 | .16 | .02 |
| Happiness | .06 | -.07 | -.06 | -.03 | -.03 | -.15 | 1 | 0 | .01 | .04 | .24 | .08 | 0 | -.02 | .02 | -.14 | -.03 | .09 | .24 | -.03 | -.01 | -.02 | -.02 | -.1 | .09 | -.01 | .03 | -.05 | 0 |
| Age | .54 | .48 | -.08 | .37 | .35 | .13 | 0 | 1 | .04 | -.33 | -.1 | -.14 | -.06 | .16 | -.03 | -.05 | -.08 | -.15 | -.19 | -.25 | -.11 | -.02 | .09 | .18 | .14 | -.05 | -.04 | -.04 | -.26 |
| Gender | -.12 | -.07 | .07 | -.14 | -.17 | .22 | .01 | .04 | 1 | .02 | .2 | -.08 | .01 | -.04 | .05 | -.01 | .08 | .01 | -.01 | .1 | -.05 | -.11 | -.08 | -.06 | 0 | -.05 | .01 | .01 | .01 |
| SD | -.15 | -.23 | .1 | -.43 | -.36 | -.12 | .04 | -.33 | .02 | 1 | .2 | .24 | .2 | -.06 | .32 | .34 | .38 | .37 | .14 | .4 | .3 | .26 | .1 | .08 | .21 | .14 | .18 | .2 | .11 |
| UN | -.24 | -.19 | .23 | -.33 | -.33 | -.07 | .24 | -.1 | .2 | .2 | 1 | .25 | .02 | -.05 | .36 | -.07 | .11 | .26 | .42 | .03 | .37 | .17 | .04 | .01 | .17 | -.09 | -.06 | .09 | .3 |
| BE | -.04 | -.11 | .04 | -.23 | -.24 | 0 | .08 | -.14 | -.08 | .24 | .25 | 1 | .32 | .26 | .33 | -.07 | .13 | .33 | .15 | .33 | .34 | .35 | .05 | .13 | .26 | .1 | .26 | .25 | .26 |
| TR | .07 | -.02 | .13 | 0 | -.03 | -.04 | 0 | -.06 | .01 | .2 | .02 | .32 | 1 | .34 | .38 | .05 | .03 | .16 | .05 | .14 | .29 | .33 | .49 | .26 | .3 | .02 | .05 | .3 | .1 |
| CO | .13 | -.01 | -.07 | .1 | .05 | .13 | -.02 | .16 | -.04 | -.06 | -.05 | .26 | .34 | 1 | .33 | .08 | .11 | 0 | -.14 | .1 | .09 | .2 | .11 | .52 | .36 | .01 | .05 | .12 | .01 |
| SE | -.2 | -.2 | .14 | -.2 | -.18 | -.11 | .02 | -.03 | .05 | .32 | .36 | .33 | .38 | .33 | 1 | .21 | .2 | .23 | .04 | .21 | .25 | .18 | .19 | .29 | .6 | .12 | .05 | .28 | .21 |
| PO | 0 | .09 | .33 | -.1 | -.05 | -.11 | -.14 | -.05 | -.01 | .34 | -.07 | -.07 | .05 | .08 | .21 | 1 | .54 | .21 | .18 | .29 | .01 | .08 | .05 | .16 | .07 | .41 | .32 | .09 | .07 |
| AC | -.09 | -.1 | .24 | -.32 | -.25 | -.12 | -.03 | -.08 | .08 | .38 | .11 | .13 | .03 | .11 | .2 | .54 | 1 | .3 | .14 | .35 | .08 | .1 | -.01 | .12 | .03 | .26 | .52 | .15 | .13 |
| HE | -.01 | 0 | .26 | -.12 | -.13 | -.02 | .09 | -.15 | .01 | .37 | .26 | .33 | .16 | 0 | .23 | .21 | .3 | 1 | .45 | .31 | .35 | .2 | .14 | .1 | .15 | .24 | .32 | .54 | .36 |
| ST | -.04 | .08 | .41 | .01 | .03 | .07 | .24 | -.19 | -.01 | .14 | .42 | .15 | .05 | -.14 | .04 | .18 | .14 | .45 | 1 | .1 | .24 | .13 | .08 | .07 | -.04 | .15 | .18 | .11 | .36 |
| SDp | -.1 | -.14 | .02 | -.2 | -.17 | .02 | -.03 | -.25 | .1 | .4 | .03 | .33 | .14 | .1 | .21 | .29 | .35 | .31 | .1 | 1 | .21 | .24 | .05 | .02 | .16 | .36 | .35 | .36 | .38 |
| UNp | -.1 | -.18 | .22 | -.14 | -.15 | -.04 | -.01 | -.11 | -.05 | .3 | .37 | .34 | .29 | .09 | .25 | .01 | .08 | .35 | .24 | .21 | 1 | .66 | .48 | .22 | .28 | -.19 | .02 | .26 | .34 |
| BEp | .1 | -.02 | .16 | -.05 | -.05 | .05 | -.02 | -.02 | -.11 | .26 | .17 | .35 | .33 | .2 | .18 | .08 | .1 | .2 | .13 | .24 | .66 | 1 | .56 | .39 | .25 | -.06 | .05 | .32 | .23 |
| TRp | .14 | .09 | .1 | -.09 | -.07 | -.01 | -.02 | .09 | -.08 | .1 | .04 | .05 | .49 | .11 | .19 | .05 | -.01 | .14 | .08 | .05 | .48 | .56 | 1 | .42 | .35 | -.07 | -.07 | .26 | .13 |
| COp | .13 | .17 | .04 | .07 | .06 | .12 | -.1 | .18 | -.06 | .08 | .01 | .13 | .26 | .52 | .29 | .16 | .12 | .1 | .07 | .02 | .22 | .39 | .42 | 1 | .49 | .12 | .19 | .3 | .07 |
| SEp | -.03 | -.09 | .09 | -.17 | -.17 | .02 | .09 | .14 | 0 | .21 | .17 | .26 | .3 | .36 | .6 | .07 | .03 | .15 | -.04 | .16 | .28 | .25 | .35 | .49 | 1 | .14 | .12 | .29 | .09 |
| POp | -.01 | .08 | .23 | .04 | .08 | .02 | -.01 | -.05 | -.05 | .14 | -.09 | .1 | .02 | .01 | .12 | .41 | .26 | .24 | .15 | .36 | -.19 | -.06 | -.07 | .12 | .14 | 1 | .67 | .34 | .33 |
| ACp | .07 | .04 | .18 | -.06 | -.02 | .06 | .03 | -.04 | .01 | .18 | -.06 | .26 | .05 | .05 | .05 | .32 | .52 | .32 | .18 | .35 | .02 | .05 | -.07 | .19 | .12 | .67 | 1 | .36 | .31 |
| HEp | .14 | .1 | .17 | .05 | .03 | .16 | -.05 | -.04 | .01 | .2 | .09 | .25 | .3 | .12 | .28 | .09 | .15 | .54 | .11 | .36 | .26 | .32 | .26 | .3 | .29 | .34 | .36 | 1 | .45 |
| STp | -.15 | -.17 | .2 | .02 | 0 | .02 | 0 | -.26 | .01 | .11 | .3 | .26 | .1 | .01 | .21 | .07 | .13 | .36 | .36 | .38 | .34 | .23 | .13 | .07 | .09 | .33 | .31 | .45 | 1 |

**Table S1**

*Correlations between all variables*

*Note.* SD: Self-direction, UN: Universalism, BE: Benevolence, TR: Tradition, CO: Conformity, SE: Security, PO: Power, AC: Achievement, HE: Hedonism, ST: Stimulation. p: perceived values of a successful entrepreneur.
